# Single-Cell Triomics Analysis of Tumor Cells Infiltrating Patient-Derived Breast Cancer Scaffolds

**DOI:** 10.1016/j.ajpath.2025.12.013

**Published:** 2026-01-22

**Authors:** Stefan Filges, Emma Jonasson, Maria Del Carmen Leiva Arrabal, Lisa Andersson, Anna Gustafsson, Dalia Dhingra, Pedro Mendez, Aik Ooi, Adam Sciambi, Göran Landberg, David Ruff, Anders Ståhlberg

**Affiliations:** ∗ Sahlgrenska Center for Cancer Research, Department of Laboratory Medicine, Institute of Biomedicine, University of Gothenburg, Gothenburg, Sweden; † Mission Bio, Inc., South San Francisco, California; ‡ Department of Clinical Genetics and Genomics, Sahlgrenska University Hospital, Region Västra Götaland, Gothenburg, Sweden; § Wallenberg Centre for Molecular and Translational Medicine, University of Gothenburg, Gothenburg, Sweden; ¶ Science for Life Laboratory, Institute of Biomedicine, University of Gothenburg, Gothenburg, Sweden

## Abstract

Cellular heterogeneity plays a critical role in tissues and diseases, including cancer. Single-cell technologies are required to provide detailed information about the phenotype and genotype of individual cells. Despite several approaches to analyzing different analytes at the single-cell level, it is challenging to assess DNA, RNA, and protein simultaneously. Here, a single-cell triomics method to assess DNA, RNA, and proteins from the same cell using a targeted sequencing approach is shown. Breast cancer cells cultured in monolayers and in patient-derived scaffolds that mimic *in vivo*–like growth conditions, both with and without chemotherapy treatment, were analyzed. Data showed that DNA, RNA, and protein biomarkers could be reliably analyzed, providing biological insights into breast cancer cell heterogeneity. In addition, chemotherapy treatment caused changes in subpopulations and expressions of biomarkers. Furthermore, cells growing in patient-derived scaffolds generated from various breast cancers affected cell heterogeneity and drug resistance differently as a result of the unique tumor-specific microenvironments. The data show that single-cell triomics provides new means to assess cancer cell heterogeneity at DNA, RNA, and protein levels.

## Graphical abstract

**Figure:**
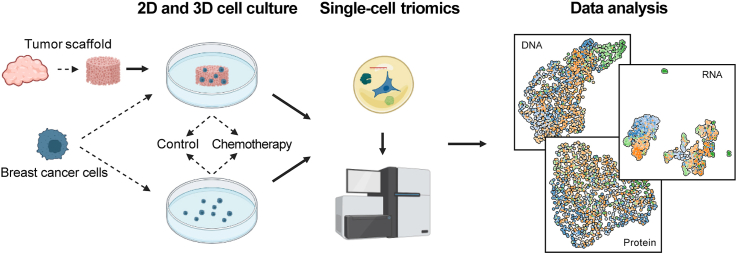

Cancer is caused by mutations that modulate different signaling pathways and biological processes.^1^ Cancer cells display large heterogeneity both between and within tumors, where intratumor heterogeneity can occur at genetical, transcriptional, and translational levels.^2^ This variability results in tumor cell subpopulations with distinct molecular and cellular characteristics, including cancer stem cell (CSC)-like cells that constitute a small, slowly proliferating and tumor-sustaining proportion of the cancer. From these, highly proliferating expansion phase cells are generated that eventually may mature into more differentiated and senescent tumor cell types. CSCs are also associated with both drug and radiotherapy resistance.^3^^,^^4^ The mutational landscape and the ability of tumor cells to acquire new mutations and cellular properties play key roles in the development of therapy resistance, determining patient outcome.^5^

Tumor cells also reside in heterogenic microenvironments that are composed of multiple tumor cell types and stromal cells such as fibroblasts, endothelial cells, and immune cells, as well as the presence of various signaling molecules. The cells interact with each other but also with the surrounding extracellular compartment, which, by its unique composition and features, drives diversity and influences cellular properties such as chemotherapy resistance, stemness, and invasive properties of the cancer cells.^6^^,^^7^ To date, it has been experimentally challenging to study tumor cell heterogeneity at cellular and molecular levels.

A plethora of single-cell methods have emerged as powerful means to identify and characterize different cell types and relate them to clinical parameters and specific biological processes. RNA sequencing, first introduced in 2009, is a widespread, advanced, and diversified technology used to molecularly profile individual cells.^8^ Today, numerous protocols for single-cell RNA sequencing with different features are available. Furthermore, several strategies to assess other analytes, including DNA,^9^ DNA methylation,^10^ chromatin accessibility,^11^ and proteins,^12^ have been developed. The optimal type of analyte to study at the single-cell level depends on several factors such as the research question, biological process of interest, and sample type. However, to fully characterize subpopulations of cells and determine molecular processes, multiple analytes often need to be analyzed, as biological processes are normally regulated at several layers and time scales, including DNA, RNA, and protein levels.

The number of established multi-omics approaches is continuously increasing^13^ and includes strategies to assess DNA and RNA,^14^^,^^15^ RNA and protein,^16^^,^^17^ RNA and DNA methylation,^18^^,^^19^ and DNA, RNA, and DNA methylation.^20^ Advanced mass spectrometry methods such as matrix-assisted laser desorption/ionization offer the possibility of studying multiple biomolecules such as RNA, DNA, lipids, proteins, and carbohydrates.^21^^,^^22^ Laboratory approaches have recently been described to assess DNA, RNA, and proteins simultaneously,^13^^,^^23^ but available options for multi-omics are still limited.

This work presents a single-cell triomics approach to assess targeted DNA, RNA, and protein simultaneously in tumor cells using microfluidics and sequencing. Selected proteins were detected by antibody-oligo conjugates, whereas cDNA and genomic DNA were targeted by sequence-specific primers. Final libraries were analyzed by using Illumina sequencing (San Diego, CA). First, two breast cancer cell lines, MCF7 and T-47D, were analyzed, both cultured in monolayers with and without 5-fluorouracil (5-FU) treatment. Next, cell-free patient-derived scaffolds (PDSs) were generated and repopulated with MCF7 cells.^6^ This experimental model system mimics *in vivo* cancer cell growth and resembles clinical properties of the original cancers and can be combined with drug testing.^24^^,^^25^ Individual MCF7 cells cultured in different PDSs with and without 5-FU treatment were profiled by using single-cell triomics. This approach and the resulting data show that single-cell triomics is feasible, which opens new means of deciphering tumor heterogeneity at different layers.

## Materials and Methods

### Cell Culture

MCF7 cells (ATCC, Manassas, VA) were cultured in Dulbecco’s modified Eagle’s medium supplemented with 10% fetal bovine serum, 1% penicillin/streptomycin, 1% l-glutamine (all Thermo Fisher Scientific, Waltham, MA), and 1% non-essential amino acids (Sigma-Aldrich, Merck, Darmstadt, Germany). T-47D cells (ATCC) were cultured in RPMI 1640 medium (Thermo Fisher Scientific) supplemented with 10% fetal bovine serum, 1% penicillin/streptomycin, 1% sodium pyruvate (Thermo Fisher Scientific), and 1% l-glutamine. Cell culture conditions were 37°C and 5% carbon dioxide in humidified atmosphere. The cell lines are regularly tested for *Mycoplasma* infection.

### Patient-Derived Scaffolds

The study protocol and amendments were approved by the regional ethical review board in Gothenburg, Sweden (515-12 and T972-18). Signed informed consent was obtained from each patient in accordance with the Declaration of Helsinki. Primary breast cancer samples were collected directly after surgery at the Clinical Pathology Diagnostic Unit at the Sahlgrenska University Hospital (Gothenburg, Sweden). Tumors from three patients were used in this study, and their histopathologic characteristics are detailed in Supplemental Table S1. The decellularization protocol for breast cancer PDSs was performed as described elsewhere.^6^

After decellularization, PDSs were cut into pieces about 6 mm in diameter using a biopsy punch needle and sliced into multiple 150 μm slices using a CM3050-S cryotome (Leica Biosystems, Nussloch, Germany). PDS slices were placed in a 48-well plate with 200 μL cell media. Then, 300,000 MCF7 cells resuspended in 500 μL of cell media supplemented with 1% antibiotic-antimycotic (Thermo Fisher Scientific) were seeded on top of each PDS slice. After 24 hours, PDSs were transferred to a new plate with fresh cell media and transferred once or twice per week to new wells to avoid cells proliferating on the plastic surface of the plate. PDS cultures were maintained for 3 weeks before downstream analysis. In total, 15 slices from each PDS were used: 10 slices were treated with 5-FU, and 5 slices remained as untreated controls.

### Drug Treatment

5-FU (Apoteket, Stockholm, Sweden) was dissolved in saline solution. For treatment of monolayer cultures, 200,000 MCF7 cells and 300,000 T-47D cells were seeded in six-well plates containing 2 mL of cell-specific media. After 24 hours, half-maximal inhibitory concentrations of 5-FU (100 μmol/L for MCF7 cells and 200 μmol/L for T-47D cells, respectively) were administered for 48 hours. For treatment of MCF7 PDS-cultured cells, 1 mmol/L 5-FU was administered for 48 hours as described elsewhere.^24^

### Single-Cell Triomics

Cells from two-dimensional cultures were washed with phosphate-buffered saline and detached with trypsin (Thermo Fisher Scientific) for 3 minutes at 37°C. PDS slices were soaked three times in phosphate-buffered saline, and cells were detached with Corning Accutase (Thermo Fisher Scientific) for 5 minutes at 37°C with gentle agitation at 180 rpm. Single-cell suspensions were then generated by using a 25-gauge needle (VWR/Avantor, Radnor, PA) and a 40 μm cell strainer (VWR). Dead cell removal was performed by using the MACS Dead Cell Removal Kit (Miltenyi Biotec, Bergisch Gladbach, Germany), according to the manufacturer’s instructions.

Triomics analysis was performed by using the Tapestri Single-Cell Multi-omics V1.1 workflow (Mission Bio, Inc., San Francisco, CA). The Tapestri platform uses microfluidics technology to combine lysed cells with barcoding beads for targeted sequencing of DNA, RNA, and antibody-oligo conjugates. The experimental procedure was performed as described previously,^26^ with a few exceptions. Briefly, single-cell suspensions containing several hundred thousand cells were stained by using the antibody-oligo conjugates targeting eight proteins (Supplemental Table S2), including mouse IgG as control; washed four times in Dulbecco’s Phosphate-Buffered Saline (Thermo Fisher Scientific); and finally resuspended to approximately 3500 cells/μL in 25% cell buffer (Mission Bio, Inc.) diluted in Dulbecco’s Phosphate-Buffered Saline. Stained cells were then encapsulated in droplets by the Tapestri instrument.

Subsequently, cell lysis, reverse transcription, and protease treatment were performed on a T-100 thermal cycler (Bio-Rad, Hercules, CA) at 50°C for 60 minutes followed by 80°C for 10 minutes. Custom DNA and RNA panels (Mission Bio, Inc.) were used. The DNA panel contained 88 amplicons targeting 68 genes, and the RNA panel targeted 25 biomarkers (Supplemental Table S2). Barcoding beads were cleaved off from targeted sequences using UV light. The target sequences were preamplified on a T-100 thermal cycler (Bio-Rad) with the following thermal program: 6 minutes at 98°C; 11 cycles of 30 seconds at 95°C, 10 seconds at 72°C, 6 minutes at 61°C, and 20 seconds at 72°C; 13 cycles of 30 seconds at 95°C, 10 seconds at 72°C, 4.5 minutes at 51°C, and 20 seconds at 72°C; and a final step of 2 minutes at 72°C. The temperature ramp-rate was 1°C per second for all steps.

After preamplification, the emulsion was broken, and libraries specific for DNA, RNA, and protein targets were enriched and purified by using Dynabeads MyOne Streptavidin (Thermo Fisher Scientific) and SPRI beads (Beckman Coulter, Brea, CA). Separate libraries for each analyte were then generated by PCR using Illumina P5 and P7 adapter primers, containing unique index sequences for demultiplexing. The library PCR used the following thermal protocol: 3 minutes at 95°C; 15 to 20 cycles of 98°C for 20 seconds, 62°C for 20 seconds, and 72°C for 45 seconds; and a final elongation at 72°C for 2 minutes. RNA libraries were amplified for 15 cycles, DNA libraries for 16 cycles, and protein libraries for 20 cycles. Final libraries were purified by using SPRI beads before quantification and quality control using capillary gel electrophoresis on a Fragment Analyzer (Agilent Technologies, Santa Clara, CA). Libraries were pooled and sequenced on either a NextSeq550 or NovaSeq (both Illumina) using 150 paired-end cycles, supplemented with 15% PhiX Control version 3 (Illumina).

### Data Preprocessing

DNA data were analyzed by using the Tapestri DNA + Protein version 2.0.2 pipeline (Mission Bio, Inc.). Briefly, adapter trimming was performed by using Cutadapt,^27^ reads with >30 nucleotides were retained, and the barcode structures were extracted. Reads were mapped to the hg19 reference genome using the BWA-MEM algorithm.^28^ Cell calling was performed using a cutoff for total DNA reads in which cells with a percentage of DNA amplicons per barcode higher than 0.2 times the mean of all DNA amplicon reads were included.

Variant calling in the DNA reads was performed by using the Genome Analysis Toolkit.^29^ Cells were genotyped by using the variant calls and cell lines identified based on their known genotypes. Read counts for single nucleotide variants and copy number variants were normalized by using centered log ratio transformation, after filtering away amplicons with less than two reads in 10% of the samples for single-nucleotide variation (SNV) or 20% of the samples for copy number variation (CNV). Normalized counts were used in dimensionality reduction with multiple analytes merged. Genetic differences between cell lines were visualized by using variant allele frequency data.

For RNA, adapter trimming was performed by using Cutadapt,^27^ reads with >30 nucleotides were retained, and the barcode structures were extracted. Reads were mapped to the hg19 reference genome using the STAR algorithm.^30^ Target reads with more than five nucleotides mapped into the intron were removed. RNA reads with cell barcodes corresponding to the cell barcodes identified as cells in the DNA library were used for downstream analysis.

Protein data were analyzed by using the Tapestri DNA + Protein version 2.0.2 pipeline. Briefly, antibody barcodes were identified, including all variants with single base substitutions. Cell barcodes were then extracted and added to the fastq header, and adapters were trimmed. Using the error-corrected map for the antibody barcodes, the antibody barcodes were identified in the reads present in the trimmed fastq file. Subsequently, the capture sequence and PCR handle were also trimmed off the reads. If the remaining read sequence was of the expected length and matched any of the antibody barcodes in the error correction map, it was accepted. Then, the number of reads for each antibody barcode within a cell was counted to generate the final output file listing the cell barcodes and the number of antibody reads for each barcode. Both RNA and protein counts were log_2_-transformed and mean-centered before analysis.

### Data Analysis

Dimensionality reduction was performed by using Uniform Manifold Approximation and Projection^31^ as implemented in the scanpy (version 1.9.1) function *sc.tl.umap*.^32^ Clustering was based on the Leiden community detection algorithm^33^ implemented in the scanpy function *sc.tl.leiden*. Differences in mean expression levels for RNA and protein assays were assessed by using Welch *t*-test implemented in the *t.test* function in R version 4.2.2 (R Foundation for Statistical Computing, Vienna, Austria). *P* values were adjusted for multiple testing using the *p.adjust* function with the Benjamini and Hochberg correction method. Results for Welch *t*-tests with Benjamini and Hochberg *post hoc* tests were considered statistically significant for adjusted *P* < 0.05. A fold change above 0.5 or below –0.5 in log_2_-scale was considered to be biologically relevant. The significance of multimodal expression was evaluated by using Hartigan dip test for unimodality^34^^,^^35^ using the *diptest* function (version 0.77-1) in R version 4.2.2. *P* values were adjusted for multiple testing as described here and considered significant for adjusted *P* < 0.05, indicating deviation from unimodality.

### Data Availability

The data sets generated and analyzed during the current study, including processed counts, are available in the National Center for Biotechnology Information’s Gene Expression Omnibus (*https://www.ncbi.nlm.nih.gov/geo/query/acc.cgi?acc=GSE291751*; accession number GSE291751).

## Results

### Single-Cell Triomics Approach

**Figure 1:**
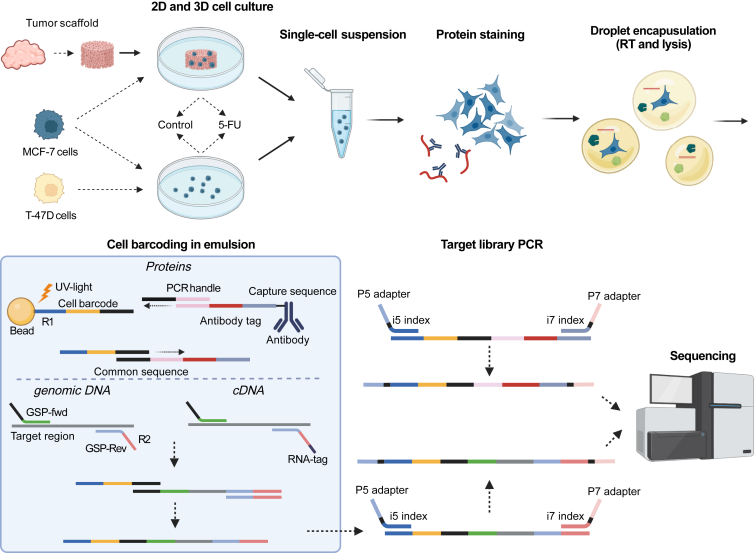
Triomics workflow. Breast cancer tumors were decellularized and MCF7 cells were cultured in three-dimensional (3D) patient-derived scaffolds (PDSs). MCF7 and T-47D cells were also grown in conventional two-dimensional (2D) cultures. Cells with and without 5-fluorouracil (5-FU) were cultured in both conditions. Single cells from the PDSs were harvested after 21 days of growth followed by 48 hours of drug treatment. Single cells were stained with antibody-oligo conjugates targeting specific proteins and subsequently encapsulated in droplets containing lysis and reverse transcription (RT) reagents. Genomic DNA, cDNA, and antibody tag sequences were barcoded in emulsion. The barcoding beads were cleaved off the sequences using UV light and the emulsion broken before isolation of the barcoded products and targeted library constructions using PCR, which introduced Illumina adapter sequences. Final libraries were purified before sequencing. GSP-fwd, gene-specific forward primer; GSP-Rev, gene-specific reverse primer; i5, Illumina index sequence 1; i7, Illumina index sequence 2; P5, P5 Illumina adapter; P7, P7 Illumina adapter; R1, read 1; R2, read 2. The figure was created with BioRender.com (Toronto, ON, Canada).

To study tumor cell heterogeneity and chemotherapy resistance in breast cancer cells, DNA, RNA, and proteins were analyzed in single cells using a modified version of the microfluidic droplet Tapestri system (Figure 1).^26^ Initially, single-cell suspensions were incubated with oligonucleotide-conjugated antibodies probing specific proteins on the cell surface. Stained cells were then encapsulated in the droplets followed by cell lysis for RNA and DNA release. In secondary droplets, cellular barcoding was performed during amplification using barcoded primers targeting the antibody-conjugated oligo-tags and the specific DNA and cDNA targets in the same single cell. Library construction was performed for each analyte after bead-based separation of the analytes. Finally, pooled libraries for all three analytes were sequenced yielding a multi-omics readout for each cell.

After data preprocessing and filtering, 22 and 65 DNA amplicons were used for SNV and CNV analysis, respectively (Supplemental Table S3). In addition, 21 RNA and seven proteins were profiled, selected based on being well-defined biomarkers for cellular proliferation, differentiation, drug resistance, epithelial–mesenchymal transition (EMT), and pluripotency in breast cancer (Supplemental Tables S3 and S4).^36^, ^37^, ^38^, ^39^, ^40^, ^41^, ^42^, ^43^, ^44^, ^45^, ^46^, ^47^, ^48^, ^49^, ^50^, ^51^, ^52^, ^53^, ^54^, ^55^, ^56^, ^57^, ^58^, ^59^, ^60^, ^61^, ^62^, ^63^

### Distinct Breast Cancer Subpopulations Defined by DNA, RNA, and Protein Data

**Figure 2:**
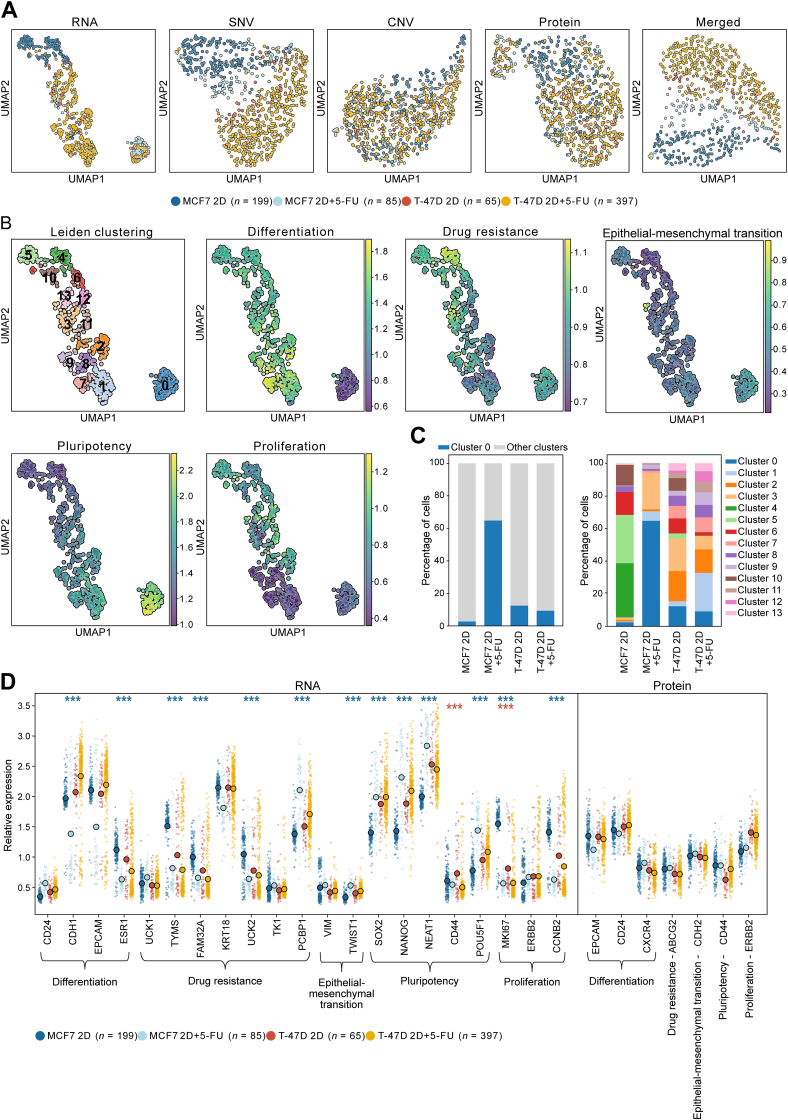
Triomics profiling of MCF7 and T-47D cells in monolayer cultures. **A:** Uniform manifold approximation and projection for dimension reduction (UMAP) analysis of MCF7 and T-47D cells with and without 5-fluorouracil (5-FU) treatment, using RNA expression, single-nucleotide variation (SNV), copy number variation (CNV), or protein expression data as well as merged RNA, SNV, CNV, and protein data. **B:** Leiden clustering based on RNA expression data shown by UMAP. Grouping resulted in 14 clusters, numbered 0 to 13. Relative expression (indicated by scale bars) of biomarker families related to differentiation, drug resistance, epithelial–mesenchymal transition, pluripotency, and proliferation biomarkers is shown. **C:** Percentage of cells based on cell type and culture condition in Leiden clusters. In the **left panel**, the percentage of cells belonging to cluster 0 in relation to any other cluster is shown. In the **right panel**, the percentage of cells per Leiden cluster is shown. **D:** Relative RNA and protein expression per gene. Circles represent mean expression. ∗∗∗*P* < 0.001, Welch *t*-test with Benjamini and Hochberg *post hoc* test, and log_2_(fold change) > |0.5|. 2D, two-dimensional.

To determine the effect of the chemotherapeutic agent 5-FU on breast cancer cell heterogeneity, the estrogen receptor α-positive breast cancer cell lines MCF7 and T-47D were cultured in monolayers with or without 48 hours of 5-FU treatment. Data were first analyzed separately for each analyte, DNA, RNA, and proteins, respectively (Figure 2A, Supplemental Figure S1, and Supplemental Table S3). At the RNA level, cells grouped clearly based on cell line type, forming distinct subpopulations. Cells also clustered according to their cell type based on SNV, whereas no clear CNV differences were observed between MCF7 and T-47D cells. At the protein level, only smaller variations were detected between the cell lines. When performing a merged analysis, combining RNA, SNV, CNV, and protein data, individual cells grouped based on their cell line identity.

Single-cell profiling using RNA as a biomarker displayed the highest degree of subclustering. Therefore, the RNA-based data were analyzed in more detail, and biomarkers were grouped together according to their tumor biological functions, including differentiation, drug resistance, EMT, pluripotency, and proliferation (Figure 2B and Supplemental Table S5). This resulted in 13 subpopulations, in which transcripts related to differentiation, pluripotency, and proliferation showed the highest regulation between identified cell groups. Clusters 4, 5, 6, and 10 were characterized by high expression of drug resistance and proliferation markers, while pluripotency markers were down-regulated; clusters 1, 7, 8, and 9 showed higher expression of pluripotency markers and lower expression of proliferation and drug resistance markers compared with the upper clusters (Supplemental Table S5). The expressions of biomarker families were gradually regulated among all 13 clusters.

The majority of MCF7 cells exposed to 5-FU (65%) grouped into cluster 0, which was the cluster with the most distinct cellular phenotype with high expression of pluripotency markers and low differentiation and proliferation, which are all features of CSCs (Figure 2C and Supplemental Table S5).^64^ 5-FU–treated MCF7 cells were also enriched in cluster 3 compared with untreated MCF7 cells, although these cells displayed no distinct cellular phenotype. Untreated MCF7 cells were mainly detected in clusters 4 (33%) and 5 (30%). T-47D cells were more evenly distributed between the clusters, where the effect of 5-FU was smaller. 5-FU–treated T-47D cells were mostly enriched in cluster 1 (24%), whereas untreated T-47D cells were more commonly observed in clusters 3 (20%) and 2 (19%).

At the RNA level, there was increased expression levels of pluripotency markers (POU5F1, NANOG, and SOX2) after exposure to 5-FU, whereas differentiation markers (CDH1 and ESR1) and proliferation markers (MKI67 and CCNA2*)* decreased in MCF7 cells (Figure 2D and Supplemental Table S6). MCF7 cells displayed an overall stronger gene regulation response to 5-FU treatment compared with T-47D cells. In addition, bimodality responses were observed in specific transcripts due to drug treatment, indicating changes in cellular phenotypes (Supplemental Figure S2 and Supplemental Table S6). For instance, the proliferation marker ERBB2 lost its bimodal distribution in 5-FU–treated MCF7 cells compared with untreated control cells. In contrast, 5-FU treatment induced bimodal expression of the differentiation markers CDH1 and EPCAM in the same cells. The EPCAM protein and RNA expression were significantly positively correlated for 5-FU–treated MCF7 and T-47D cells (Supplemental Figure S3). However, the bimodality observed at the RNA level was not conserved at the protein level. Protein and RNA expression of ERBB2 was positively correlated in untreated MCF7 and T-47D cells but not in 5-FU–treated cells. Interestingly, correlation between RNA and protein expression for the pluripotency marker CD44 could only be observed in 5-FU–treated MCF7 cells but not for T-47D cells. In T-47D cells, CD44 was significantly down-regulated upon 5-FU treatment at the RNA level while expression increased at the protein level.

### Breast Cancer Cells Adapt to the Extracellular Microenvironment in a Patient-specific Manner

**Figure 3:**
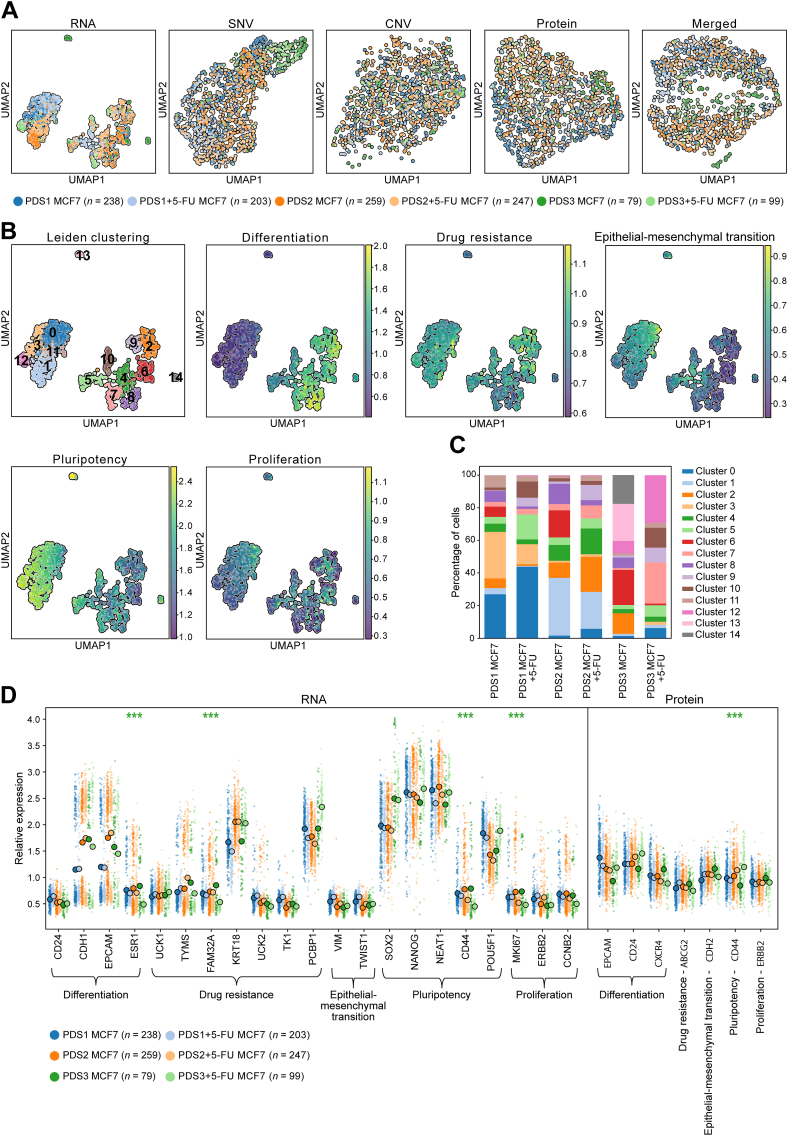
Triomics profiling of MCF7 cells cultured in patient-derived scaffolds (PDSs). **A:** Uniform manifold approximation and projection for dimension reduction (UMAP) analysis of MCF7 cells cultured in three different PDSs with or without 5-fluorouracil (5-FU) treatment, using RNA expression, single-nucleotide variation (SNV), copy number variation (CNV), or protein expression as well as merged SNV, CNV, protein, and RNA data. **B:** Leiden clustering based on RNA expression data shown by UMAP. Grouping resulted in 15 clusters, numbered 0 to 14. Relative expression (indicated by scale bars) of biomarker families related to differentiation, drug resistance, epithelial–mesenchymal transition, pluripotency, and proliferation biomarkers is shown. **C:** Percentage of cells based on cell type and culture condition in relation to Leiden clusters. **D:** Relative RNA and protein expression per gene. Circles represent mean expression. ∗∗∗*P* < 0.001, Welch *t*-test with Benjamini and Hochberg *post hoc* test, and log_2_(fold change) > |0.5|.

To study breast cancer cells in an *in vivo*–like microenvironment with its unique structural components, PDSs were used as an experimental model system.^6^ Three different primary breast cancers were decellularized by using mild detergent washes to remove cellular debris and patient DNA. Clinico-pathologic properties of the breast cancers are shown in Supplemental Table S1. The decellularized PDSs were recellularized with MCF7 cells. After 3 weeks of culture, the recellularized PDSs were treated with 5-FU for 48 hours before single-cell analysis using the triomics workflow (Figure 1).^24^ Each analyte was analyzed separately as well as merged for each PDS (PDS1 to PDS3) with and without 5-FU treatment (Figure 3A and Supplemental Table S7).

As for two-dimensional cell cultures, the RNA data revealed most distinct subpopulations. Interestingly, MCF7 cells from PDS3 were distinguished from cells growing in the two other PDSs also based on SNV data, despite all scaffolds being reintroduced with the same homogeneous MCF7 cells. This finding indicates clonal expansion during PDS recellularization because it was observed in both 5-FU–treated and untreated cells (Figure 3A). However, no clear distinctions could be made between cells and PDSs based on CNV or protein data.

Subgrouping based on RNA data showed that clusters 0, 1, 3, 11, and 12 formed a larger group consisting of 44% of all cells, with reduced expression of differentiation markers as well as higher expression of EMT and pluripotency markers (Figure 3B and Supplemental Table S8). In contrast, the other main group (clusters 2 and 4-10) showed increased levels of differentiation markers and lower expression of EMT and pluripotency. Two small clusters (13 and 14) with high expression of pluripotency markers were also identified with cells from untreated PDS3. Furthermore, there were clusters that were systematically enriched for 5-FU–treated (5, 9, and 10) and untreated (6 and 8) cells for all three PDSs (Figure 3C and Supplemental Table S8).

Interestingly, PDS-specific clusters could also be identified, including clusters 0, 3, and 11 for PDS1 distinguished by generally higher levels of the EMT markers TWIST1 and VIM, as well as pluripotency markers NEAT1 and POU5F1 (Supplemental Table S8). Clusters 1 and 4 were enriched in cells from PDS2 and showed high expression of pluripotency markers and differentiation markers, respectively. Cells from PDS3 were mainly found in cluster 7, showing increased levels of the differentiation markers CDH1 and EPCAM, and in cluster 12, with enrichment of drug resistance marker PCBP1, as well as the pluripotency markers NEAT1 and POU5F1. Finally, co-regulation of RNA expression appeared more similar between cells from the two estrogen receptor–negative PDSs (PDS1 and PDS3) when treated (Supplemental Figure S4). Collectively, the data show that specific PDSs with their unique microenvironments induced distinct cellular phenotypes.

When analyzing individual RNAs and proteins, fewer markers were significantly differently expressed between 5-FU–treated and untreated cells compared with the monolayer-cultured MCF7 cells (Figure 3D and Supplemental Table S9). Here, only three RNA transcripts (ESR1, FAM32A, and MKI67) and one protein (CD44) met the applied cutoffs for significance, specifically for cells grown in PDS3 (Figure 3D). Although the magnitude of mean expression differences was not pronounced for most RNAs and proteins, similar patterns of regulation could be observed between different PDSs upon treatment (Supplemental Figure S5). For instance, the pluripotency markers NANOG and SOX2 showed significant bimodal expression in untreated cells for both PDS2 and PDS3, which changed to a unimodal pattern in both cases upon treatment. The differentiation marker CD24 was negatively correlated between RNA and protein level in both PDS1 and PDS2 without and with treatment, whereas EPCAM RNA and protein expression showed a positive correlation in untreated PDS1 and PDS3 samples that disappeared upon drug treatment (Supplemental Figure S6).

## Discussion

The first single-cell triomics study for DNA, RNA, and protein was reported in 2012 with quantitative PCR as readout.^65^ Here, the use of single-cell triomics to assess DNA, RNA, and proteins using targeted sequencing was shown in an experimental model system that provides an *in viv*o–like tumor microenvironment. Numerous studies have reported the benefits of single-cell analysis compared with conventional cell bulk analyses.^66^^,^^67^ A remaining technical challenge in single-cell analysis is to assess multiple analytes in the same single cell. Here, DNA, RNA, and protein were simultaneously analyzed to improve the capability to decipher cellular phenotypes. For instance, DNA mutations are irreversible and usually occur over time, whereas the cellular response at the RNA and protein levels occurs on different time scales to various perturbations.^5^^,^^20^ Integrating DNA, RNA, and protein data derived from the same single cell thus offers the possibility of harnessing the synergistic effect of using multiple biomarkers representing different facets of a cell’s genotype as well as phenotype.

The applied triomics approach is targeted, which is a limitation in studies aiming at identifying new biomarkers. However, a targeted approach can be beneficial in terms of cost and sensitivity when detecting selected biomarkers. An improved sensitivity is possible because each assay can be optimized and sequenced in-depth. A related approach for analyzing DNA, RNA, and protein in the same cells combines high-throughput RNA analysis with targeted genome analysis and protein assessment using index sorting with fluorescence-activated cell sorting.^23^ Ultimately, future triomics strategies should allow for complete and integrated genome, transcriptome, and proteome analyses for more high-throughput options. Here, the DNA panel was based on common mutations in cancer while RNA and protein targets were carefully selected based on reported properties related to cellular proliferation, differentiation, drug resistance, EMT, and pluripotency in breast cancer (Supplemental Table S4); all of these factors are relevant when studying chemotherapy-treated breast cancer cells.

A key element for cancer cells is the surrounding microenvironment, including the noncellular extracellular matrix and non-tumor cells, influencing cellular phenotypes as well as aggressive properties of the tumor such as drug resistance.^68^^,^^69^ Growing cells in PDS cultures compared with monolayer cultures has been shown to change the cellular phenotype, by decreasing expression of proliferation- and differentiation-related biomarkers, while increasing the expression levels of EMT- and pluripotency-related markers, suggesting an increase of cells with CSC features.^6^^,^^70^

PDSs are unique in their protein composition and hence affect cancer cells differently in a tumor-specific manner that is directly correlated to clinical parameters such as tumor grade or patient age.^70^^,^^71^ Therefore, this model system can be useful in many types of preclinical studies, including drug testing.^72^ This study showed reliable detection of DNA, RNA, and protein in cells detached from PDS samples, indicating that the applied triomics method is applicable also to more complex samples. Furthermore, the expression pattern between PDS cultures varied as a consequence of the PDSs originating from three different primary breast cancers. In future studies, it would be interesting to include a larger cohort of PDS samples to be able to further investigate the connection to clinical parameters. Data showed that all analytes provide relevant information and that the cellular heterogeneity increased when cells were allowed to grow in an *in vivo*–like environment.

5-FU treatment generally enhances the CSC-phenotype of cancer cells,^73^ and each PDS has been shown to influence drug responses in a manner specific to the tumor microenvironment.^7^ In the performed experiments, 5-FU–treated MCF7 cells in monolayer cultures induced a higher fraction of CSC-like cells, whereas the effect in PDSs was more complex, despite using a higher drug concentration. Generally, cancer cells become more resistant to drug treatments in experimental three-dimensional models due to limited accessibility to the drug but also as a result of cancer cells adapting to each other and extracellular structural components. For example, cell proliferation is less pronounced in three-dimensional cultures than in monolayer cultures, generating more diversified cellular phenotypes.^72^^,^^74^^,^^75^ Potentially, a longer treatment time would have had a greater effect on the infiltrating tumor cells in this more complex model system.

Interestingly, variations in SNVs could be observed between MCF7 cells cultured in different PDSs, showing that the microenvironment may influence cellular properties and heterogeneity. In contrast, 5-FU treatment generated no mutations in the applied experimental settings, most likely due to the short exposure time. In this study, the RNA biomarkers were more informative than the proteins. The reasons may be that the selected proteins are less informative than the RNA biomarkers, or simply because fewer proteins were analyzed. The correlation between expression levels of RNA and protein for the same gene can vary between experimental settings,^76^^,^^77^ signifying the value of analyzing both analytes simultaneously. In addition, in some cases, the RNA expression, but not the protein expression, showed a bimodal pattern, highlighting differences between the two analytes. Clustering of cells from PDSs based on RNA expression profiles revealed both PDS-specific clusters as well as drug treatment–induced clusters. However, further analysis revealed that the PDS-induced effect was larger on the cultured cells than the effect of the 5-FU treatment for these three specific PDSs, despite the evident influence of the drug. In summary, the biological value of analyzing multiple biomarkers at the DNA, RNA, and protein level simultaneously in the same single cells was shown.

This study shows that single-cell triomics is feasible and can be applied to complex cell culture conditions, including multiple cell lines, drug treatments, and relevant three-dimensional model systems. Panels of targeted DNA, RNA, and protein assays can be designed and validated for various single-cell applications, providing biological and clinical information that otherwise is challenging to obtain.

## Disclosure Statement

A.St. is board member and declares stock ownership in Tulebovaasta and Simsen Diagnostics and stock ownership in Iscaff Pharma. G.L. is a board member and declares stock ownership in Iscaff Pharma. Iscaff Pharma holds interests and patent related to the PDS technology (patent numbers P3535421B9 and US11840732B2). D.D., P.M., A.O., and D.R. were employees of Mission Bio, Inc. during these experiments and are co-inventors of “Method and apparatus for simultaneous targeted sequencing of DNA, RNA and protein” (US Patent number 11365441). A.Sc. is employed by Mission Bio, Inc. S.F. is employed by Simsen Diagnostics.

## Supplemental Data

Supplemental Figure S1 Mutation signatures for monolayer cells. Heatmap of variant allele frequencies (VAF) for mutations observed in MCF7 and T-47D cells in monolayer cultures without or with 5-fluorouracil (5-FU) treatment. **Gray boxes** indicate missing data. 2D, two-dimensional.

Supplemental Figure S2 Bimodality in RNA and protein expression in monolayer cells. Violin plots show the distributions of expressions for cells without or with 5-fluorouracil (5-FU) treatment in MCF7 and T-47D cells. A **dashed line** is inserted to separate RNA and protein data. Hartigan dip test with Benjamini and Hochberg *post hoc* test was used to test for bimodality. ∗*P* < 0.05, ∗∗*P* < 0.01, ∗∗∗*P* < 0.001.

Supplemental Figure S3 Correlation between RNA and protein expression in monolayer cells. Scatter plots show the relationship between RNA and protein expression for EPCAM, CD44, ERBB2, and CD24 in MCF7 and T-47D cells cultured in monolayers, without or with 5-fluorouracil (5-FU) treatment. Spearman correlation coefficients (*r*) and *P* values are shown in each plot.

Supplemental Figure S4 Correlation heatmaps of RNA expression in patient-derived scaffold (PDS)-cultured cells. Heatmaps showing the pair-wise correlations between specific RNA biomarkers in MCF7 cultured in three different PDSs, without or with 5-fluorouracil (5-FU). Spearman correlation coefficients are shown.

Supplemental Figure S5 Bimodality in RNA and protein expression in patient-derived scaffold (PDS)-cultured cells. Violin plots show the distributions of expressions for cells without or with 5-fluorouracil (5-FU) treatment in MCF7 PDSs. A **dashed line** is inserted to separate RNA and protein data. Hartigan dip test with Benjamini and Hochberg *post hoc* test was used to test for bimodality. ∗*P* < 0.05, ∗∗*P* < 0.01, ∗∗∗*P* < 0.001.

Supplemental Figure S6 Correlation between RNA and protein expression in patient-derived scaffold (PDS)-cultured cells. Scatter plots show the relationship between RNA and protein expression for CD24, CD44, EPCAM, and ERBB2 in MCF7 cells cultured in PDSs without or with 5-fluorouracil (5-FU) treatment. Spearman correlation coefficients (*r*) and *P* values are shown in each plot.
