# Supplementary figures and images for: Single-Cell Triomics Analysis of Tumor Cells Infiltrating Patient-Derived Breast Cancer Scaffolds
Source: Am J Pathol. 2026 Jan 22;196(4):1016–27. doi: 10.1016/j.ajpath.2025.12.013 (PMC13084604; doi:10.1016/j.ajpath.2025.12.013)

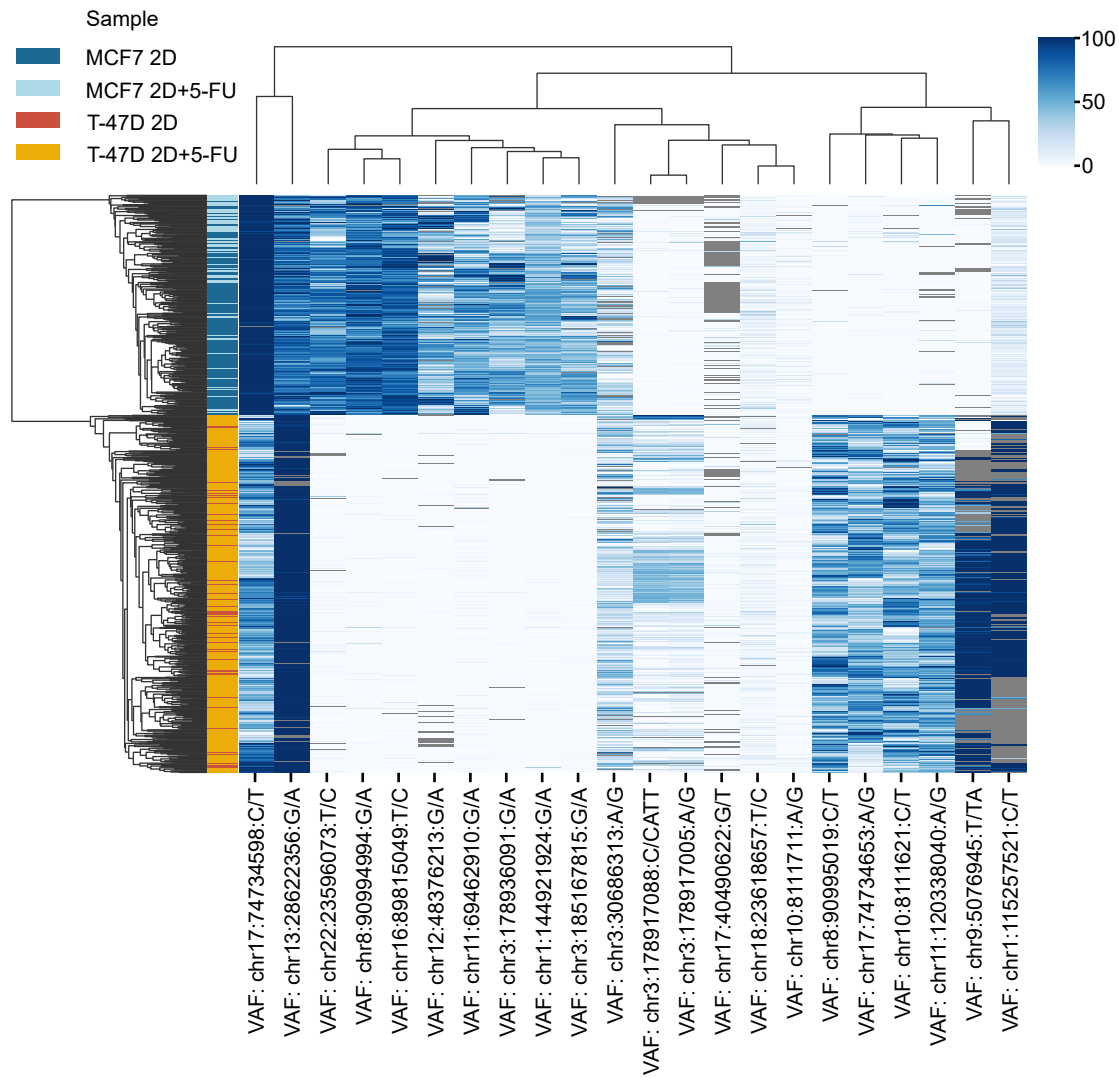

Supplement: Supplemental Figure S1 — Mutation signatures for monolayer cells. Heatmap of variant allele frequencies (VAF) for mutations observed in MCF7 and T-47D cells in monolayer cultures without or with 5-fluorouracil (5-FU) treatment. Gray boxes indicate missing data. 2D, two-dimensional. [file mmc1.pdf]

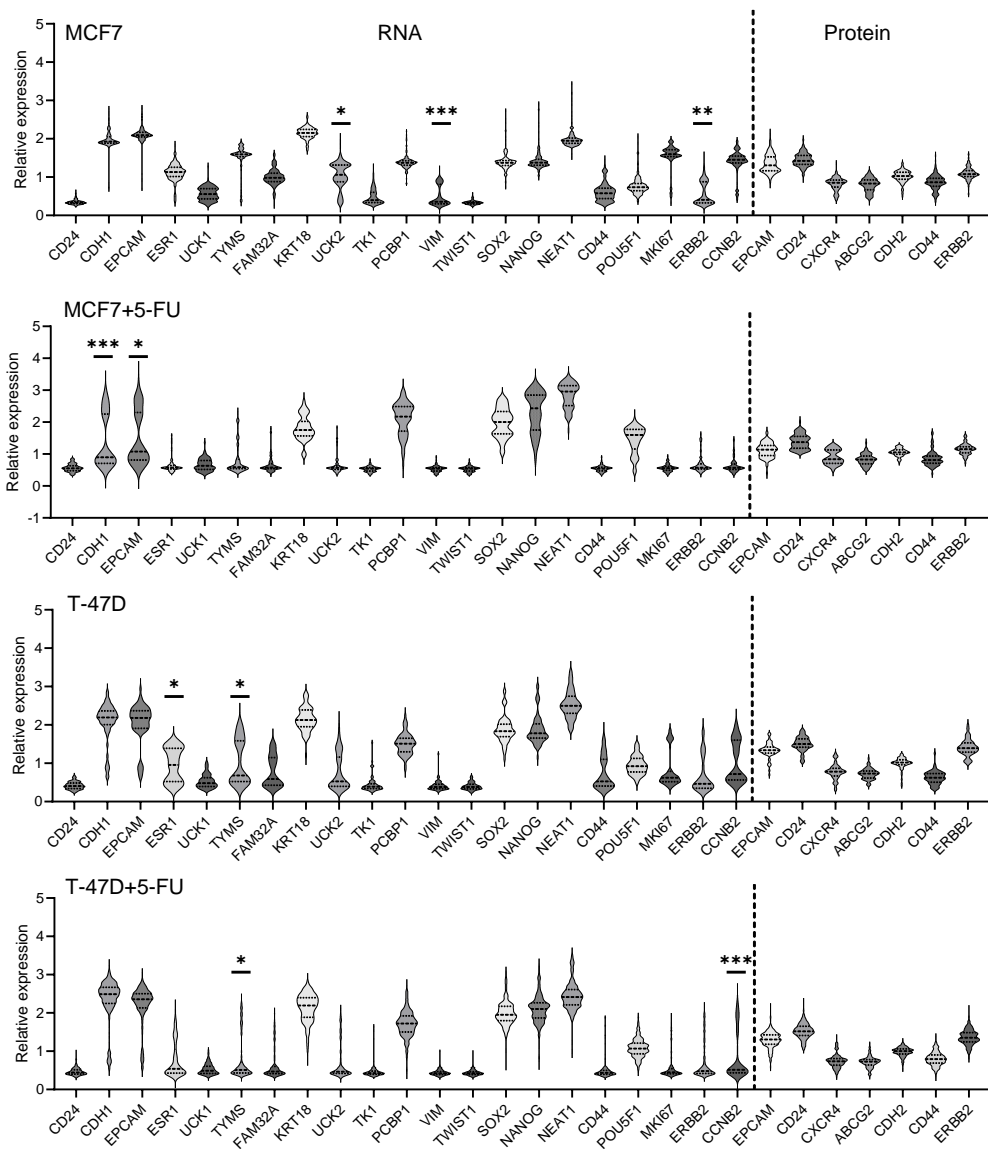

Supplement: Supplemental Figure S2 — Bimodality in RNA and protein expression in monolayer cells. Violin plots show the distributions of expressions for cells without or with 5-fluorouracil (5-FU) treatment in MCF7 and T-47D cells. A dashed line is inserted to separate RNA and protein data. Hartigan dip test with Benjamini and Hochberg post hoc test was used to test for bimodality. ∗P < 0.05, ∗∗P < 0.01, ∗∗∗P < 0.001. [file mmc2.pdf]

MCF7

MCF7+5-FU

T-47D

T-47D+5-FU

EPCAM

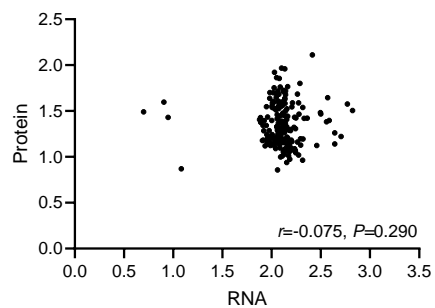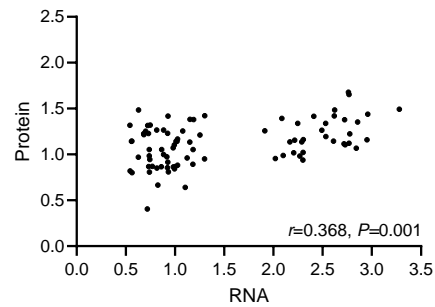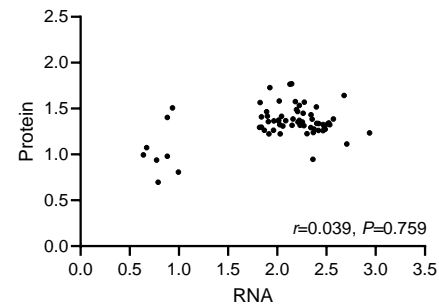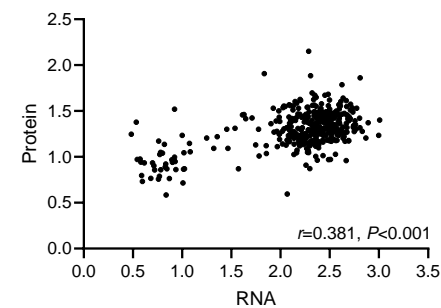

CD44

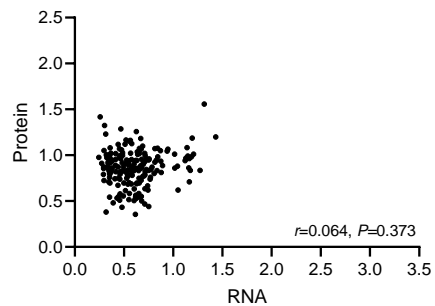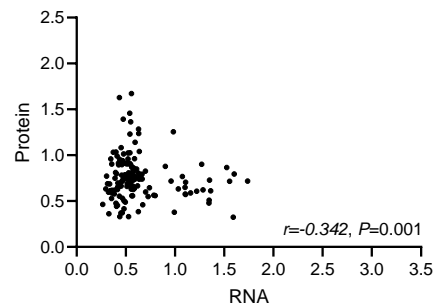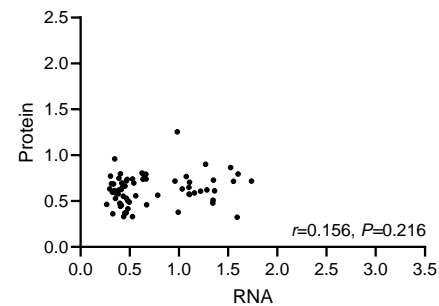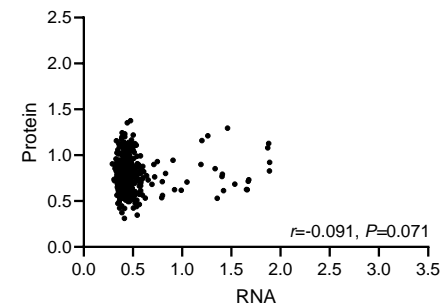

ERBB2

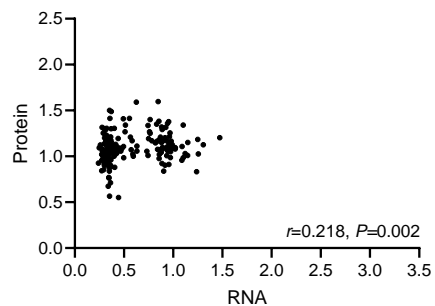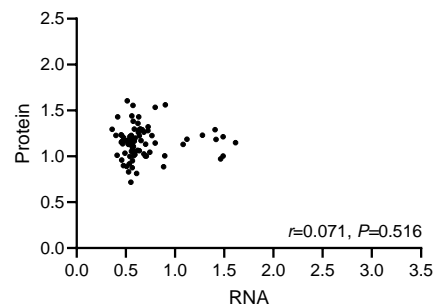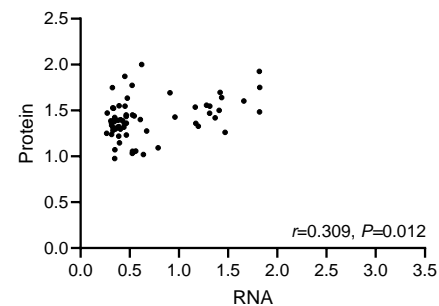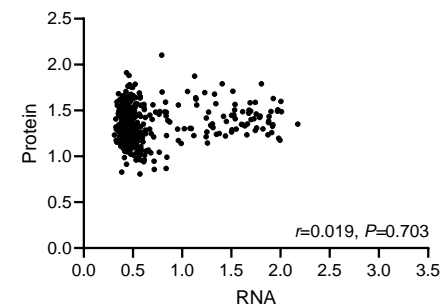

CD24

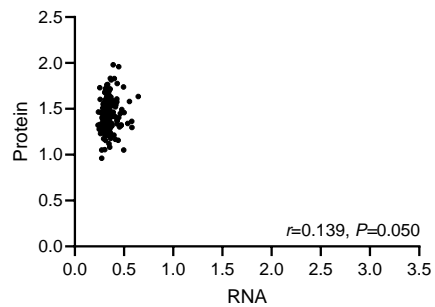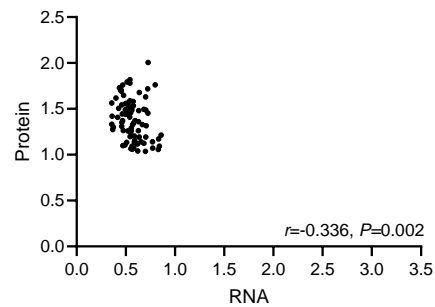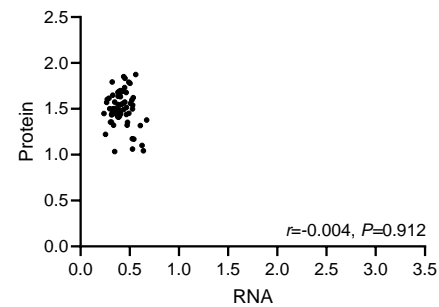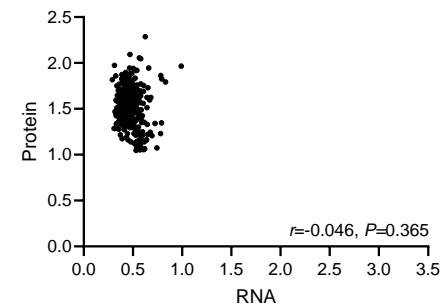

Supplement: Supplemental Figure S3 — Correlation between RNA and protein expression in monolayer cells. Scatter plots show the relationship between RNA and protein expression for EPCAM, CD44, ERBB2, and CD24 in MCF7 and T-47D cells cultured in monolayers, without or with 5-fluorouracil (5-FU) treatment. Spearman correlation coefficients (r) and P values are shown in each plot. [file mmc3.pdf]

PDS 1

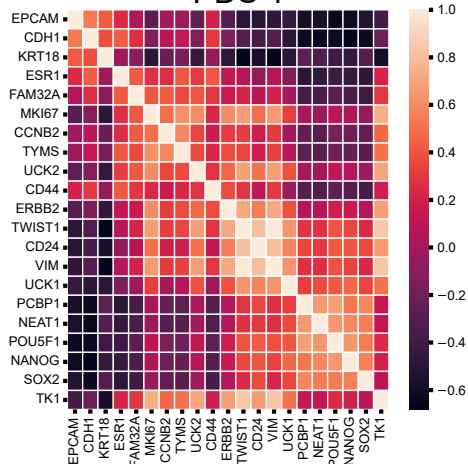

PDS 2

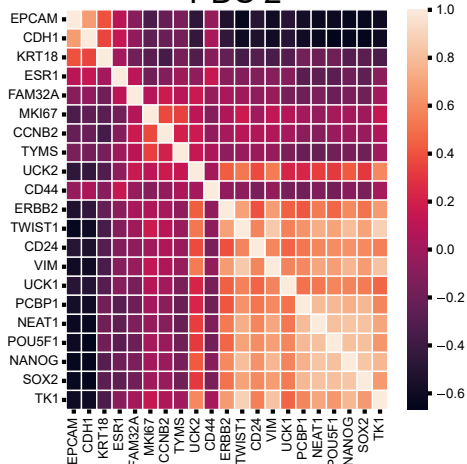

PDS 3

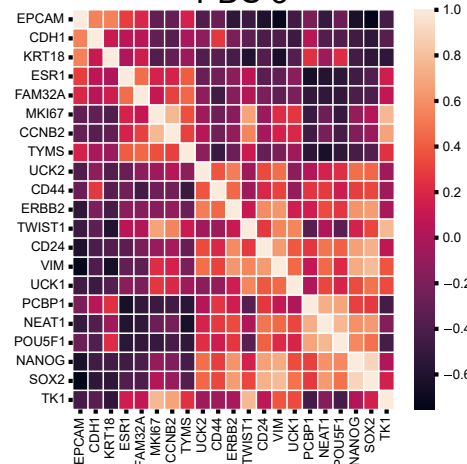

PDS 1 + 5-FU

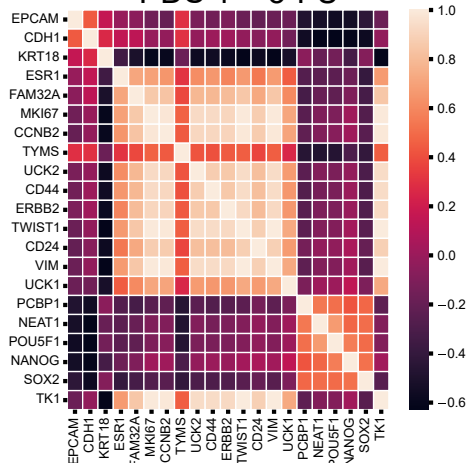

PDS 2 + 5-FU

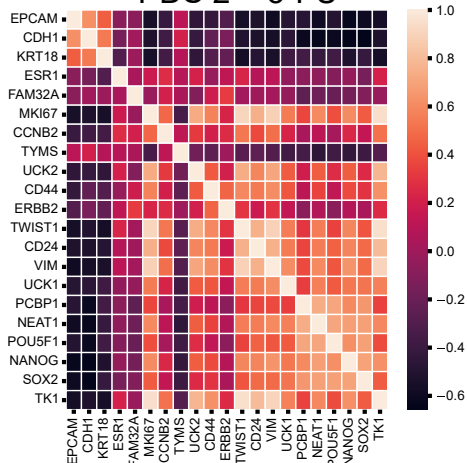

PDS 3 + 5-FU

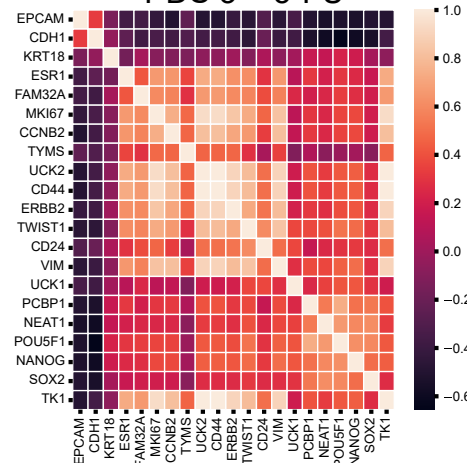

Supplement: Supplemental Figure S4 — Correlation heatmaps of RNA expression in patient-derived scaffold (PDS)-cultured cells. Heatmaps showing the pair-wise correlations between specific RNA biomarkers in MCF7 cultured in three different PDSs, without or with 5-fluorouracil (5-FU). Spearman correlation coefficients are shown. [file mmc4.pdf]

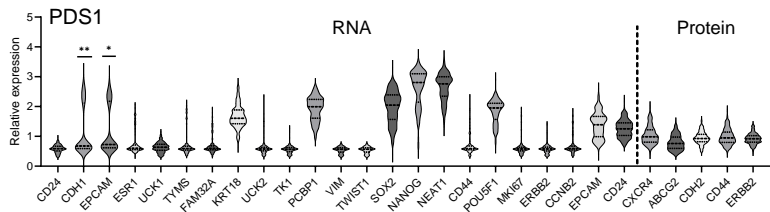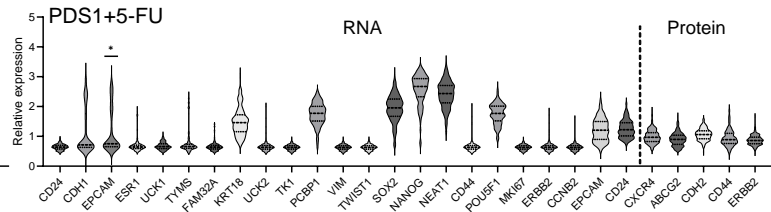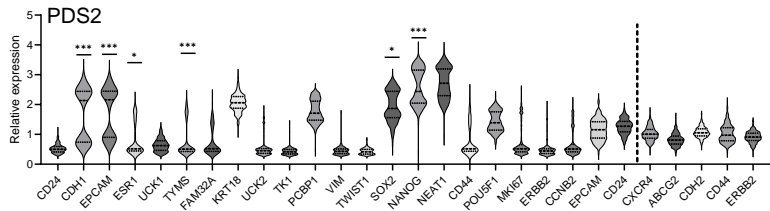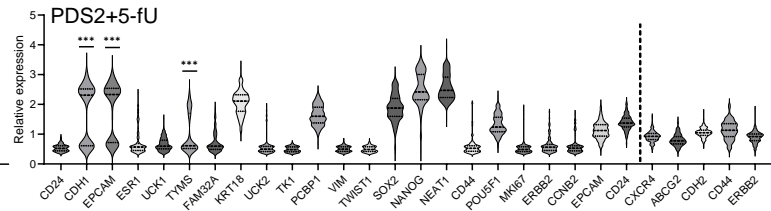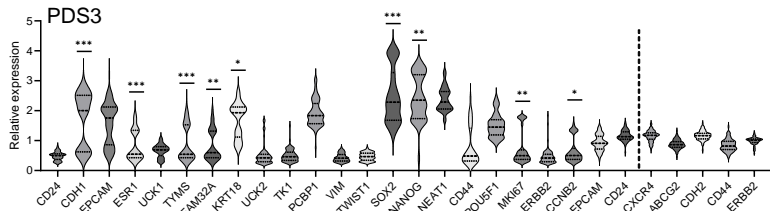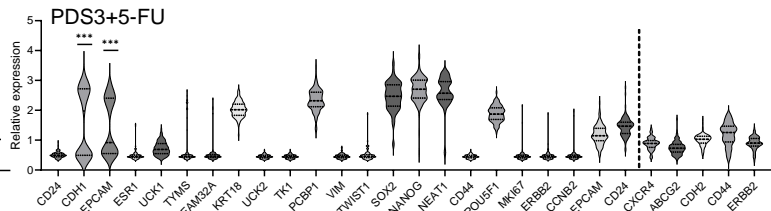

Supplement: Supplemental Figure S5 — Bimodality in RNA and protein expression in patient-derived scaffold (PDS)-cultured cells. Violin plots show the distributions of expressions for cells without or with 5-fluorouracil (5-FU) treatment in MCF7 PDSs. A dashed line is inserted to separate RNA and protein data. Hartigan dip test with Benjamini and Hochberg post hoc test was used to test for bimodality. ∗P < 0.05, ∗∗P < 0.01, ∗∗∗P < 0.001. [file mmc5.pdf]

## CD24

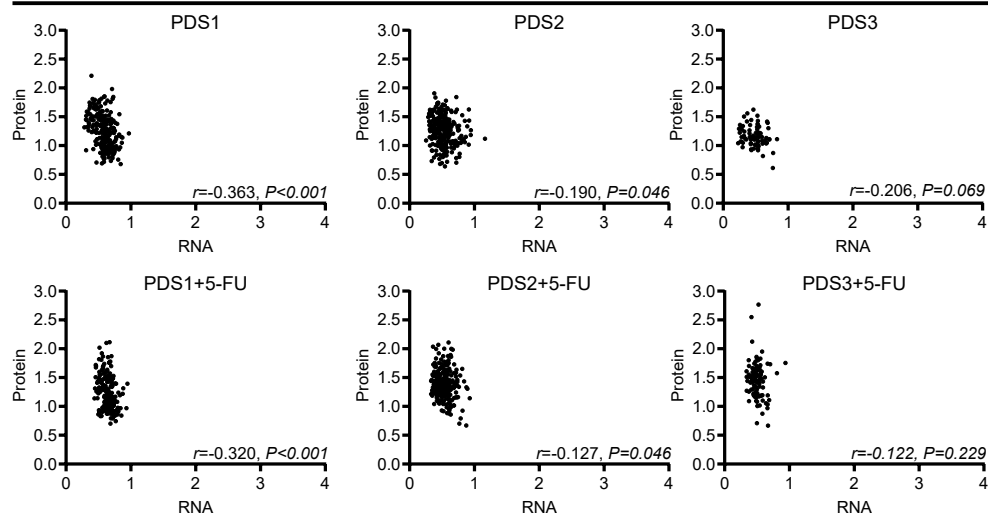

## CD44

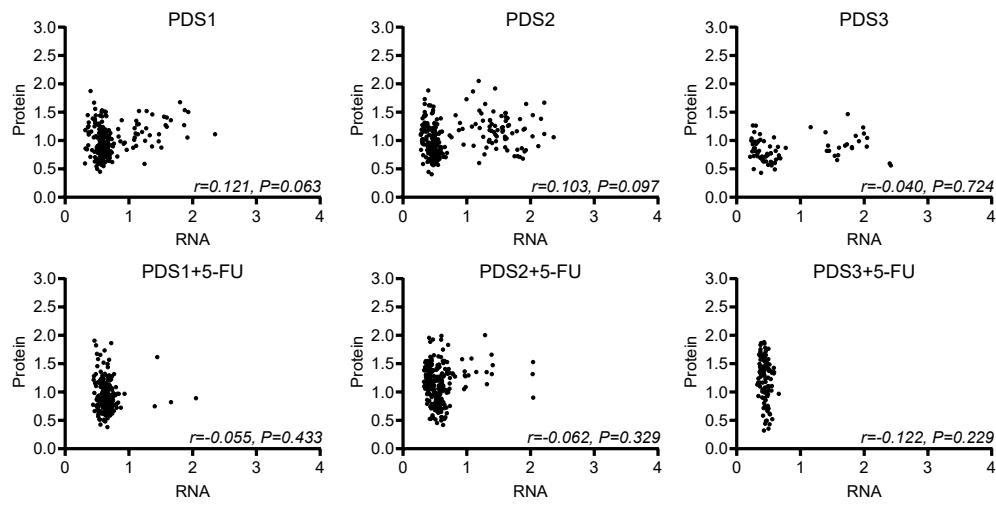

## EPCAM

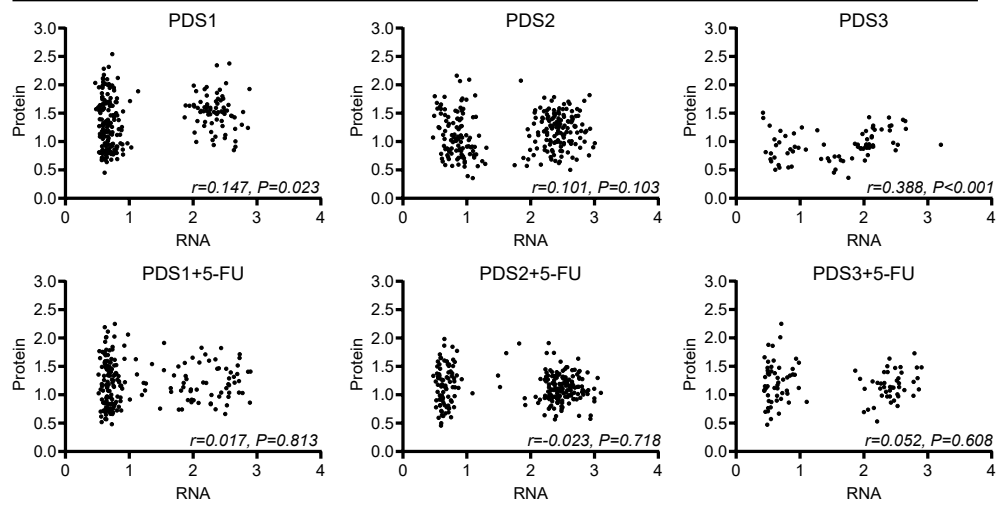

## ERBB2

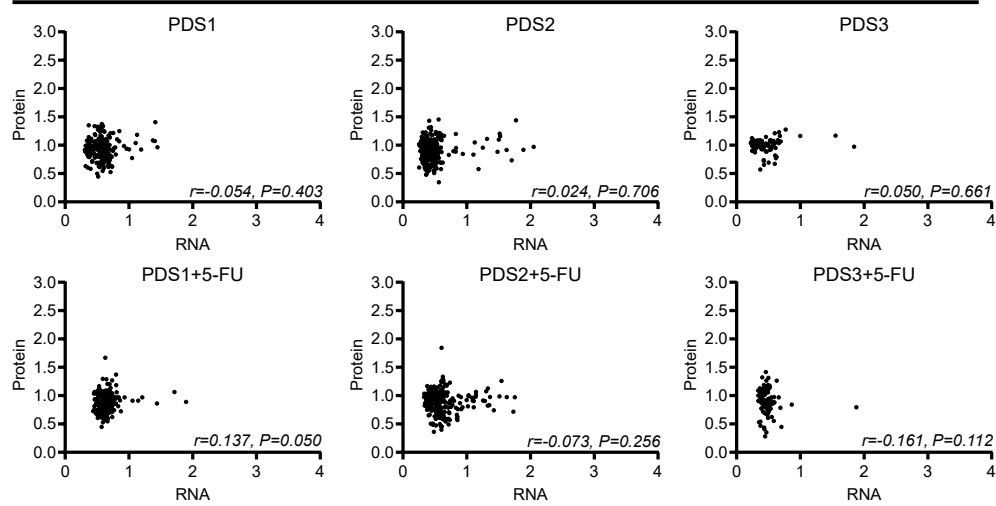

Supplement: Supplemental Figure S6 — Correlation between RNA and protein expression in patient-derived scaffold (PDS)-cultured cells. Scatter plots show the relationship between RNA and protein expression for CD24, CD44, EPCAM, and ERBB2 in MCF7 cells cultured in PDSs without or with 5-fluorouracil (5-FU) treatment. Spearman correlation coefficients (r) and P values are shown in each plot. [file mmc6.pdf]
